# On two species of *Phintella* Strand, 1906 from Hainan, China (Araneae, Salticidae)

**DOI:** 10.3897/BDJ.12.e138400

**Published:** 2024-11-20

**Authors:** Cheng Wang, Jiahui Gan, Xiaoqi Mi

**Affiliations:** 1 Guizhou Provincial Key Laboratory for Biodiversity Conservation and Utilization in the Fanjing Mountain Region, Tongren University, Tongren, China Guizhou Provincial Key Laboratory for Biodiversity Conservation and Utilization in the Fanjing Mountain Region, Tongren University Tongren China

**Keywords:** Chrysillini, DNA barcode, morphology, Island, taxonomy

## Abstract

**Background:**

In our recent examination of the *Phintella* specimens collected from Hainan Tropical Rainforest National Park, a new species and the unknown female of *P.liae* Wang, Mi & Peng, 2023 were recognised, based on the morphological characteristics and molecular evidence.

**New information:**

A new species of *Phintella* Strand, 1906 is described: *P.hongkan* sp. nov. (♂♀) from Hainan, China. The unknown female of *P.liae* Wang, Mi & Peng, 2023 is also described for the first time. Diagnostic photos of both species are provided.

## Introduction

*Phintella* Strand, 1906, one of the most species-rich genera of the tribe Chrysillini Simon, 1901, is represented by 82 nominal species widely distributed mainly from the Oriental and Palaearctic Regions (Maddison 2015, World Spider Catalog 2024). Like most salticid genera, the taxonomic study of the genus is restricted by a high rate (more than 34%) of single-sex known species. Moreover, species are diverse in habitus and copulatory organs, indicating it should not be monophyletic and the generic position of some current members needs further revision. To date, 32 species have been recorded from China, of which 21 are endemic (World Spider Catalog 2024). The goal of the present work is to describe a new *Phintella* species and the unknown female of *P.liae* Wang, Mi & Mi, 2023.

## Materials and methods

Specimens were collected by beating shrubs. They were preserved in absolute ethanol. Specimens are deposited in Tongren University (TRU) in Tongren, China. Methods of specimen examination, observation and photo generation followed Wang et al. (2024). A partial fragment of the mitochondrial cytochrome oxidase subunit I (COI) gene of the two species was amplified and sequenced using the primers COI-TY-F1 and COI-TY-R1 (Yamasaki et al. 2018). The pairwise genetic distances (Kimura two-parameter [K2P]) (Table 1) were calculated using MEGA 6.0 to assess the genetic differences.

All measurements are given in millimetres. Leg measurements are given as: total length (femur, patella, tibia, metatarsus, tarsus). Abbreviations used in the text and figures are as follows: **AERW** anterior eye row width; **AME** anterior median eye; **ALE** anterior lateral eye; **BP** basal epigynal plate; **CD** copulatory duct; **CO** copulatory opening; **E** embolus; **EFL** eye field length; **FD** fertilisation duct; **PERW** posterior eye row width; **LP** lamellar process; **PL** posterior tegular lobe; **PLE** posterior lateral eye; **PME** posterior median eye; **RTA** retrolateral tibial apophysis; **S** spermatheca; **TB** tegular bump.

## Taxon treatments

### Phintella hongkan

Wang, Gan & Mi sp. nov.

2BB192CD-24BD-57BC-9FAA-FCFA353132D6

1AA6B662-CB96-4D26-ADAA-CA36EBDC3277

#### Materials

- **Type status:** Holotype. **Occurrence:** individualID: TRU-JS 0825; sex: male; associatedSequences: GenBank: PQ412689; occurrenceID: 1D552E33-339E-527F-BA02-612228678928; **Taxon:** scientificName: *Phintellahongkan* sp. nov.; **Location:** country: China; stateProvince: Hainan; county: Baisha Li Autonomous; locality: Yuanmen Township, around of Hongkan Waterfall; verbatimElevation: 565 m; verbatimLatitude: 19°4.94′N; verbatimLongitude: 109°30.03′E; **Identification:** identifiedBy: Cheng Wang; **Event:** samplingProtocol: Beating shrubs; year: 2024; month: 09; day: 17
- **Type status:** Paratype. **Occurrence:** individualID: TRU-JS 0826; individualCount: 1; sex: female; associatedSequences: GenBank: PQ412690; occurrenceID: 6CF37AE7-5727-53CB-B8BB-022C5CAD1556; **Taxon:** scientificName: *Phintellahongkan* sp. nov.; **Location:** country: China; stateProvince: Hainan; county: Baisha Li Autonomous; locality: Yuanmen Township, around of Hongkan Waterfall; verbatimElevation: 565 m; verbatimLatitude: 19°4.94′N; verbatimLongitude: 109°30.03′E; **Identification:** identifiedBy: Cheng Wang; **Event:** samplingProtocol: Beating shrubs; year: 2024; month: 09; day: 17

#### Description

**Figure 1.**
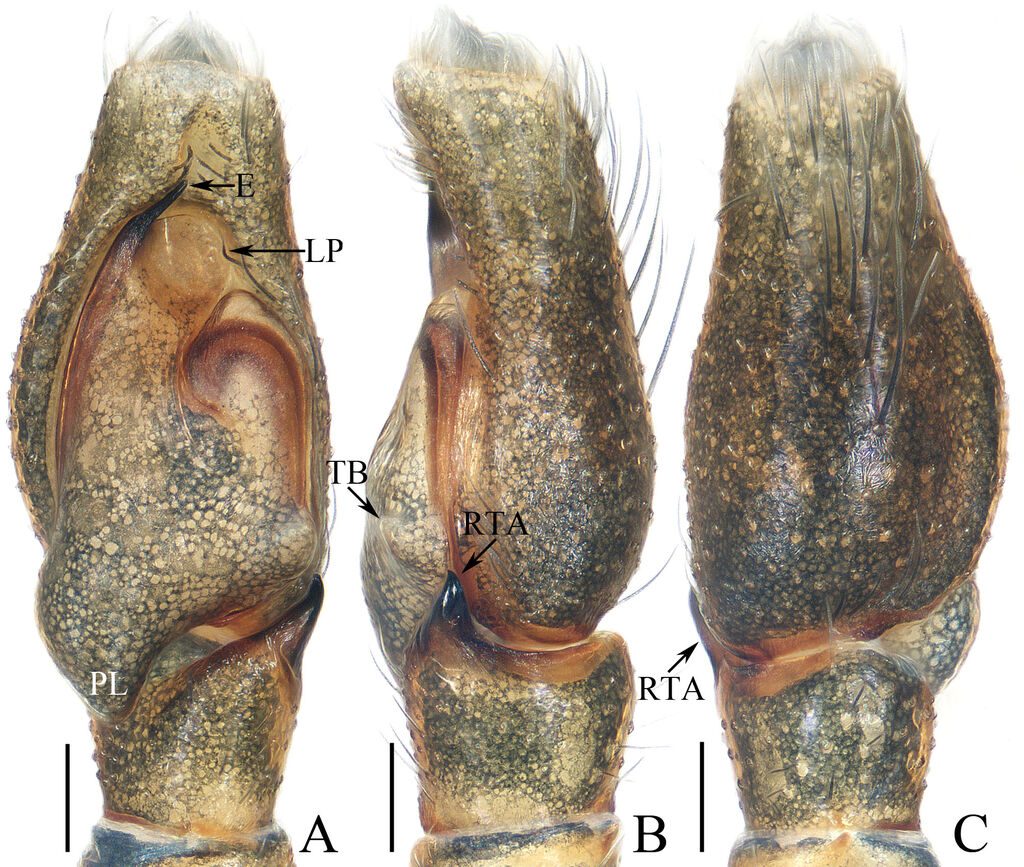
Male palp of *Phintellahongkan* sp. nov., holotype (TRU-JS 0825). **A** ventral; **B** retrolateral; **C** dorsal. Scale bars: 0.1 mm.

**Figure 2.**
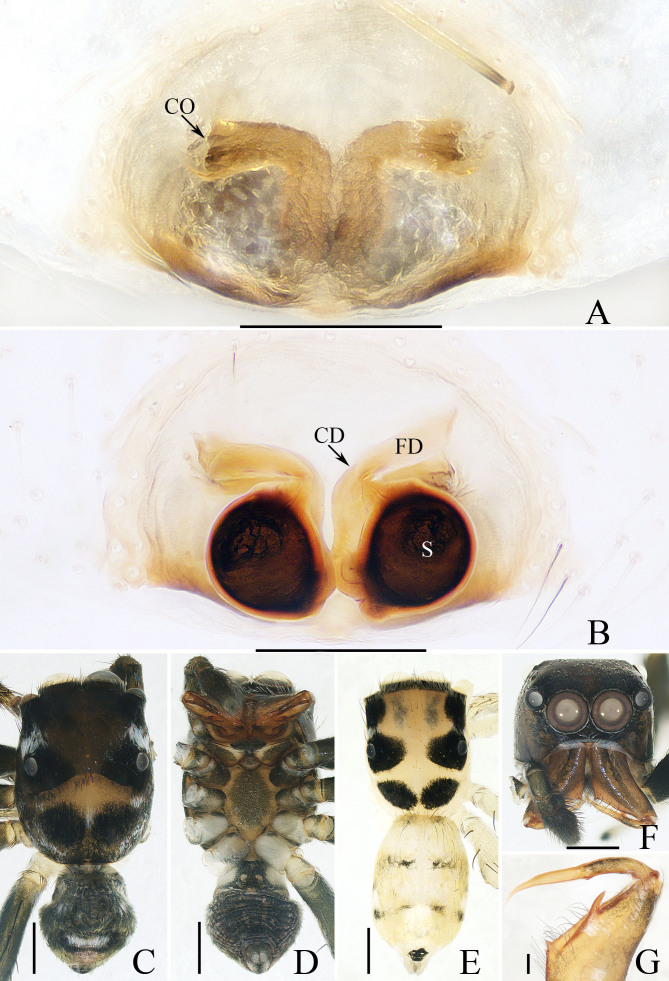
*Phintellahongkan* sp. nov. **A**, **B**, **E** female paratype (TRU-JS 0826) and **C**, **D**, **F**, **G** holotype (TRU-JS 0825). **A** epigyne, ventral; **B** vulva, dorsal; **C**, **E** habitus, dorsal; **D** ditto, ventral; **F** carapace, frontal; **G** chelicera, posterior. Scale bars: 0.1 mm (**A**, **B**, **G**); 0.5 mm (**C**-**F**).

**Male** (Fig. 1, Fig. 2C, D, F and G). Total length 2.93. Carapace 1.74 long, 1.39 wide. Abdomen 1.26 long, 0.98 wide. Eye sizes and inter-distances: AME 0.44, ALE 0.22, PME 0.21, AERW 1.26, PERW 1.24, EFL 0.83. Legs: I 4.21 (1.25, 0.60, 1.13, 0.83, 0.40), II 3.54 (1.10, 0.48, 0.88, 0.75, 0.33), III 4.14 (1.25, 0.53, 0.95, 1.03, 0.38), IV 4.44 (1.30, 0.53, 1.05, 1.13, 0.43). Carapace elevated, dark brown to dark, covered with clusters of white scale-like setae between PLEs and PMEs, with pair of sub-triangular dark patches inner to PMEs, irregular median yellow area and pair of oval dark patches bearing dense dark scale-like setae posteriorly on thorax; fovea dark red, longitudinal. Chelicerae dark yellow, mingled with green-brown, with two promarginal teeth and one larger, medially located retromarginal tooth. Endites sub-square, bearing scopulae on antero-inner portions. Labium tapered. Sternum yellow to green-brown. Legs green-brown to dark brown, except metatarsi and tarsi III and IV pale. Abdomen almost oval, dorsum setose, with median, transverse white setal band; venter dark.

Palp (Fig. 1A-C): tibia slightly wider than long in retrolateral view; retrolateral tibial apophysis strongly sclerotised, tapered, with pointed tip slightly curved ventrally; cymbium about 1.8 times longer than wide; bulb elongated; posterior lobe posteriorly extended, with blunt end; tegular bump sub-triangular, near distal portion of retrolateral tibial apophysis; lamellar process anteriorly located, near half-round; embolus short, strongly sclerotised, originating from antero-prolateral portion of bulb, slightly curved at base and with rather blunt end.

**Female** (Fig. 2A, B and E). Total length 3.01. Carapace 1.48 long, 1.15 wide. Abdomen 1.61 long, 1.07 wide. Eye sizes and inter-distances: AME 0.39, ALE 0.20, PME 0.18, AERW 1.13, PERW 1.13, EFL 0.78. Legs: I 2.71 (0.85, 0.43, 0.63, 0.50, 0.30), II missing, III 3.16 (1.00, 0.43, 0.65, 0.75, 0.33), IV 3.56 (1.08, 0.40, 0.83, 0.90, 0.35). Carapace (Fig. 2E) pale to yellow, with similar dark patches as in male. Chelicerae yellow, with two promarginal teeth and one retromarginal tooth almost equal in size. Endites coloured as chelicerae. Labium pale. Legs pale, spiny. Abdomen elongate-oval, dorsum (Fig. 2E) pale to yellow, with two discontinuous, transverse dark stripes; venter pale, with terminal green-brown patch.

Epigyne (Fig. 2A and B): wider than long; copulatory openings mediolaterally located on atrium and opened laterally; copulatory ducts transversely extended at origin and then downward descending, distal end connected to base-inner portion of spermathecae; spermathecae oval, apart from each other by about one-fourth their width; fertilisation ducts lamellar.

#### Diagnosis

The male of *Phintellahongkan* sp. nov. resembles that of *P.arcuata* Huang, Wang & Peng, 2015 in having similar habitus and palpal structure, but it differs in: 1) the carapace posteriorly has a pair of dark patches (Fig. 2C) versus absent in *P.arcuata* (Huang et al. 2015: fig. 1A); 2) the cheliceral fang lacks terminal flap (Fig. 2G) versus present in *P.arcuata* (Huang et al. 2015: figs. 1B and 3C); 3) the posterior lobe is downward extended (Fig. 1A) versus postero-retrolaterally in *P.arcuata* (Huang et al. 2015: figs. 1C and 3A); 4) the bulb has antero-retrolaterally extended lamellar process (Fig. 1A) versus retrolaterally in *P.arcuata* (Huang et al. 2015: figs. 1C and 3A); 5) the retrolateral shoulder of bulb has an inverted V-shaped edge (Fig. 1A) versus arc-shaped edge *in P.arcuata* (Huang et al. 2015: figs. 1C and 3A). The female closely resembles that of *P.pygmaea* (Wesołowska, 1981) in having an almost identical epigyne, but it can be easily distinguished by the absence of the basal epigynal plate (Fig. 2A) versus present *in P.pygmaea* (Wang et al. 2023: fig. 25A) and the presence of a pair of dark patches posteriorly on carapace (Fig. 2E) versus absent in *P.pygmaea* (Wang et al. 2023: fig. 25E).

#### Etymology

The specific name is after Hongkan Waterfall, a famous scenic spot nears the type locality; noun (name) in apposition.

#### Distribution

Known only from the type locality in Hainan, China.

### Phintella liae

Wang, Mi & Peng, 2023

19E7EAD6-3424-5962-8898-C315E3C69240

#### Materials

- **Type status:** Other material. **Occurrence:** individualID: TRU-JS 0827; individualCount: 1; sex: male; associatedSequences: GenBank: PQ412691; occurrenceID: 17184FC9-EE41-590B-90DA-FC6B50E5F51A; **Taxon:** scientificName: *Phintellaliae* Wang, Mi & Peng, 2023; **Location:** country: China; stateProvince: Hainan; county: Baisha Li Autonomous; locality: Yuanmen Township, around of Hongkan Waterfall; verbatimElevation: 565 m; verbatimLatitude: 19°4.94′N; verbatimLongitude: 109°30.03′E; **Identification:** identifiedBy: Cheng Wang; **Event:** samplingProtocol: beating shrubs; year: 2024; month: 9; day: 17
- **Type status:** Other material. **Occurrence:** individualID: TRU-JS 0828; individualCount: 1; sex: female; associatedSequences: GenBank: PQ412692; occurrenceID: AFD822E1-CBAC-5194-8ABB-DDA635EF866A; **Taxon:** scientificName: *Phintellaliae* Wang, Mi & Peng, 2023; **Location:** country: China; stateProvince: Hainan; county: Baisha Li Autonomous; locality: Yuanmen Township, around of Hongkan Waterfall; verbatimElevation: 565 m; verbatimLatitude: 19°4.94′N; verbatimLongitude: 109°30.03′E; **Identification:** identifiedBy: Cheng Wang; **Event:** samplingProtocol: beating shrubs; year: 2024; month: 9; day: 17

#### Description

**Figure 3.**
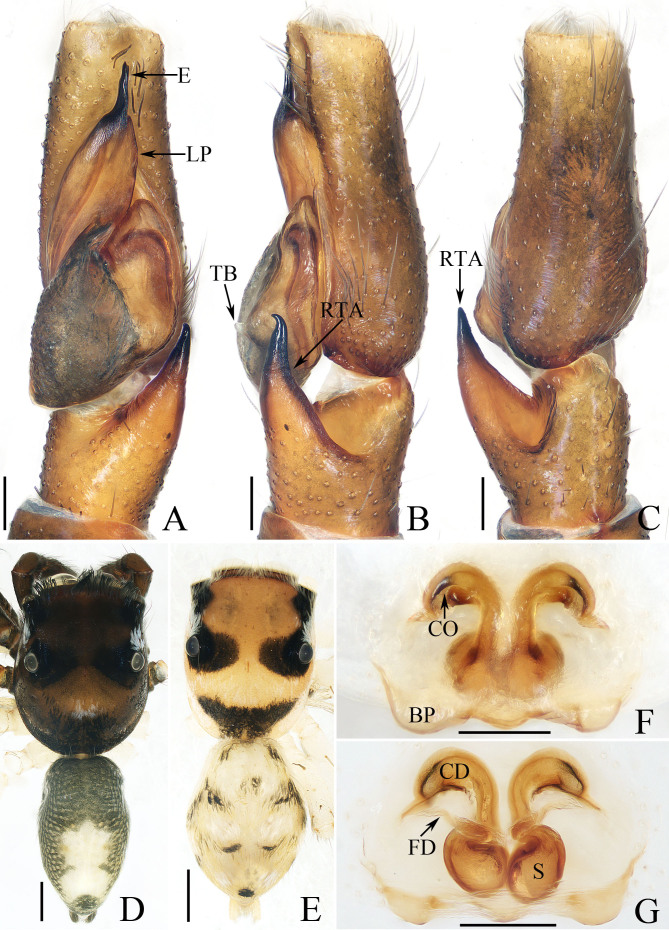
*Phintellaliae* Wang, Mi & Peng, 2023, **A**-**D** male (TRU-JS 0827) and **E**-**G** female (TRU-JS 0828). **A** palp, ventral; **B** ditto, retrolateral; **C** ditto, dorsal; **D**, **E** habitus, dorsal; **F** epigyne, ventral; **G** vulva, dorsal. Scale bars: 0.1 mm (**A**-**C**, **F**, **G**); 0.5 mm (**D**, **E**).

**Male** (Fig. 3A-D). See Wang et al. (2023).

**Female** (Fig. 3E-G). Total length 3.15. Carapace 1.59 long, 1.28 wide. Abdomen 1.60 long, 1.15 wide. Eye sizes and inter distances: AME 0.41, ALE 0.22, PME 0.21, AERW 1.24, PERW 1.25, EFL 0.82. Legs: I 2.59 (0.68, 0.48, 0.63, 0.50, 0.30), II 2.44 (0.68, 0.45, 0.58, 0.43, 0.30), III 3.01 (0.88, 0.40, 0.68, 0.70, 0.35), IV 3.69 (1.13, 0.48, 0.88, 0.85, 0.35). Carapace yellow to dark yellow, covered with clusters of white scale-like setae between PLEs and PMEs, with pair of dark patches inner to PMEs and sub-triangular dark patch posteriorly on thorax; fovea red, longitudinal. Chelicerae red yellow, with two promarginal teeth and one retromarginal tooth. Endites sub-square, with scopulae on antero-inner portions. Labium darker than endites. Sternum almost oval, with straight anterior margin. Legs pale yellow. Abdomen oval, dorsum mainly yellow, with white and dark scale-like setal stripes; venter pale.

Epigyne (Fig. 3F and G): wider than long, with broad basal epigynal plate with two lateral protrudings; copulatory openings anteriorly located, almost slit-shaped; copulatory ducts curved into C-shape at proximal and then downward descending to connect to ventro-median portions of spermathecae; spermathecae oval, touched; fertilisation ducts lamellar.l

#### Diagnosis

The male was thoroughly diagnosed in Wang et al. (2023). The female of this species resembles that of *P.dives* (Simon, 1899) in having a similar epigyne, but it can be easily distinguished by the distance between protrudings of basal epigynal plates, which is about three-fifths the epigynal width (Fig. 3F) versus less than half of the epigynal width in *P.dives* and by the presence of anterior curved portions of spermathecae (Fig. 3G) versus absent in *P.dives* (see the drawings of Prószyński (1984)).

#### Distribution

China (Guangxi, Hainan).

## Supplementary Material

XML Treatment for Phintella hongkan

XML Treatment for Phintella liae

## Figures and Tables

**Table 1.** transpecific and interspecific nucleotide divergences for two *Phintella* species using Kimura two parameter model.

Species	TRU-JS 0825	TRU-JS 0826	TRU-JS 0827	TRU-JS 0828
*P.hongkan* TRU-JS 0825				
*P.hongkan* TRU-JS 0826	0.009			
*P.liae* TRU-JS 0827	0.127	0.125		
*P.liae* TRU-JS 0828	0.135	0.134	0.009	
